# Emergency procedural pathway combined with graded zoning management is associated with improved in-hospital survival and quality of life in patients with acute myocardial infarction

**DOI:** 10.3389/fmed.2026.1841718

**Published:** 2026-07-06

**Authors:** Xiaoyan Song, Ying Ma, Yadong Shang, Changchang Zhao, Renli Cheng

**Affiliations:** Department of Emergency, The Affiliated Suzhou Hospital of Anhui Medical University, Suzhou, Anhui, China

**Keywords:** acute myocardial infarction, emergency procedural pathway, graded zoning management, in-hospital survival, quality of life

## Abstract

**Background:**

Acute myocardial infarction (AMI) requires rapid emergency care; however, triage delays in high-volume emergency departments remain a persistent challenge. Evidence on the combination of emergency procedural pathways with graded zoning management in AMI care is limited.

**Methods:**

This prospective quasi-experimental study used sequential pre-post enrollment and included 126 patients with AMI. Patients admitted during the routine-care phase (CG; *n* = 64) received routine emergency nursing care, and patients admitted after protocol implementation (IG; *n* = 62) received an emergency procedural pathway combined with graded zoning management. Because enrollment was non-randomized and non-concurrent, all effect estimates were interpreted as associations rather than causal effects.

**Results:**

In-hospital survival was higher in the IG (96.8%, 60/62) than the CG [81.3%, 52/64; risk difference 15.5%, 95% CI 4.7–26.4%; *p* = 0.008; adjusted odds ratio [aOR] 6.50, 95% CI 1.32–32.0, *p* = 0.022]. First-aid time was shorter (67.30 ± 3.90 vs. 75.70 ± 4.50 min; mean difference −8.40, 95% CI −9.87 to −6.93; *p* < 0.001), and hospital stay reduced (14.62 ± 2.05 vs. 18.08 ± 4.55 days; mean difference −3.46, 95% CI −4.69 to −2.23; *p* < 0.001). Standardized reperfusion intervals were also shorter in the IG (door-to-balloon time among primary-PCI patients 78.5 ± 12.3 vs. 92.4 ± 15.6 min; door-to-needle time among thrombolysis patients 32.5 ± 6.8 vs. 41.2 ± 8.9 min; both *p* < 0.001). The cohort was predominantly STEMI (83.9% IG, 82.8% CG), with comparable reperfusion-modality use between groups. MACE was lower (6.5% vs. 18.8%; aOR 0.30, 95% CI 0.09–0.97, *p* = 0.045). Quality-of-life scores improved across all GQOL-74 domains (all *p* < 0.001); post-intervention SAS (31.70 ± 6.85 vs. 38.05 ± 7.24; *p* < 0.001) and SDS (34.10 ± 3.20 vs. 36.28 ± 3.18; *p* < 0.001) were lower in the IG. Patient satisfaction was higher (87.1% vs. 65.6%; risk difference 21.5%, 95% CI 7.4% to 35.6%; *p* = 0.005).

**Conclusion:**

In this single-center quasi-experimental study, the emergency procedural pathway combined with graded zoning management was associated with higher in-hospital survival, shorter treatment-related time, fewer cardiovascular complications, and better discharge quality-of-life and satisfaction scores. The magnitude of the observed mortality difference should be interpreted cautiously because residual confounding from unmeasured AMI severity, reperfusion eligibility, and temporal changes cannot be excluded.

## Introduction

Acute myocardial infarction (AMI) is one of the most serious and critical manifestations of coronary heart disease (1). This condition arises when the coronary arteries experience persistent ischemia and hypoxia, leading to disruptions in myocardial cell function and ultimately resulting in myocardial cell necrosis. The most common clinical manifestations of AMI are rapid onset and sudden onset chest pain. Most patients present with pain in the posterior chest or precordial area at onset, often accompanied by a sense of impending death (2). Without prompt treatment, AMI can seriously threaten patient survival. Relevant studies have shown that high-intensity exercise, significant emotional fluctuations, and long-term smoking and alcohol consumption can trigger cardiovascular events, precipitating AMI (3). Epidemiological data further indicate that the incidence of AMI is increasing annually, and its high mortality rate has placed a considerable burden on patients and their families (4).

The emergency departments of most hospitals in China receive a large volume of patients throughout the year. The intermingling of emergency and non-emergency patients often extends waiting times for patients with AMI, potentially delaying effective treatment for critically ill individuals who most urgently require it (5). Therefore, timely, scientific, and effective rescue measures are essential. These measures include the rapid collection of subjective and objective patient information, assessment of conditions, and simultaneous measurement of vital signs, all of which are needed to obtain reliable clinical data, conserve time and resources, and improve survival to discharge while reducing AMI mortality rates.

International guidelines and chest pain center programs have established that structured pathways minimizing door-to-reperfusion time improve outcomes in ST-segment elevation myocardial infarction (STEMI), and similar protocolized approaches have been associated with shorter ischemic time and reduced complications (6, 7). Recent STEMI studies further reinforce the clinical importance of time-sensitive emergency care. Söner and Özbek reported that incomplete ST-segment resolution after primary PCI was associated with adverse outcomes in STEMI, highlighting the importance of effective reperfusion and downstream myocardial recovery (8). In a separate cohort, Söner et al. found that emergency department delay time was an independent predictor of 1-year all-cause mortality among STEMI patients undergoing primary PCI, although the association with in-hospital mortality was not statistically significant (9). These findings support the rationale for evaluating emergency workflows that reduce early treatment delays, while also emphasizing that outcome differences in observational pathway studies must be interpreted in light of baseline severity and reperfusion-related factors. Beyond individual cohort studies, organized systems of care provide the most robust evidence that time-focused emergency processes improve survival in STEMI. Hospital-level strategies that compress door-to-balloon time (10), coordinated statewide and regional reperfusion networks (11), and population-based analyses showing that shorter health-system delay is associated with lower mortality (12) have collectively established systems-of-care optimization as a central determinant of outcome in acute myocardial infarction. This body of work provides the conceptual foundation for the integrated emergency workflow evaluated in the present study.

In this study, a combined approach of emergency procedural pathway and graded zoning management was evaluated to examine its association with in-hospital survival and quality of life among patients with AMI, with the aim of optimizing the treatment process, strengthening the sense of responsibility and risk awareness of medical staff, and improving patient satisfaction.

## Methods

### Study design and setting

This was a prospective quasi-experimental study with a sequential pre-post implementation design conducted at the Department of Emergency, Suzhou Municipal Hospital affiliated to Anhui Medical University, Suzhou, Anhui Province, China. The study enrolled patients from January 2022 to July 2023. Patients admitted during the first phase of the study period (January 2022 to October 2022) received routine emergency nursing and constituted the control group (CG), while patients admitted during the second phase (November 2022 to July 2023), after implementation of the new nursing protocol, received emergency procedural pathway combined with graded zoning management and constituted the intervention group (IG). The sequential design was selected to avoid contamination within a single emergency department after staff training and pathway activation had been introduced. Nevertheless, because the groups were enrolled in different calendar periods rather than concurrently, the design is inherently vulnerable to temporal confounding from changes in staffing, operator experience, case mix, equipment availability, and ancillary protocols. This issue was considered during interpretation and is explicitly addressed in the limitations.

This study was approved by the Ethics Committee of Suzhou Municipal Hospital, affiliated with Anhui Medical University. The requirement for written informed consent was waived by the Ethics Committee because the intervention represented a department-level workflow optimization and the analysis used de-identified clinical data collected during routine care. This waiver is concordant with the consent statement recorded in the journal's ethics declaration; accordingly, no written informed consent for research participation was sought from any patient. The only consent referenced elsewhere in this manuscript is the procedural (treatment) consent obtained for invasive or thrombolytic therapy as part of routine emergency clinical care, which is distinct from research consent and was managed under standard institutional emergency procedures. For patients requiring urgent treatment, all clinical decisions and treatment-related permissions were obtained according to institutional emergency-care procedures and applicable national regulations. This study was conducted in accordance with the principles of the Declaration of Helsinki.

### Participants

A total of 126 patients diagnosed with acute myocardial infarction (AMI) were enrolled as research subjects according to the predefined inclusion and exclusion criteria. All eligible patients were included in each study phase.

A priori sample size estimation was performed based on the primary outcome of in-hospital survival until discharge. Assuming a survival rate of 80% in the control group and an expected rate of 95% in the intervention group, with a two-sided α of 0.05 and a statistical power of 80%, a minimum of 56 patients per group was required for the study. To account for potential dropouts and incomplete data, a target enrollment of at least 60 patients per group was established. The assumed control-group survival of 80% was based on the department's pre-implementation clinical experience with patients with acute myocardial infarction managed under routine emergency nursing during the period preceding the study, whereas the anticipated intervention-group survival of 95% reflected the magnitude of improvement reported for optimized emergency and fast-track reperfusion pathways in comparable acute coronary syndrome populations (13, 14). These figures were treated as planning estimates for powering the study rather than as anticipated effect sizes for inference.

### Inclusion criteria

(1) Patients met the diagnostic criteria for AMI according to institutional diagnostic standards and contemporary guideline-based definitions (15, 16), with elevated cardiac biomarkers (troponin T and myoglobin above the normal range) and ECG changes compatible with AMI, including persistent ST-segment elevation, inverted T waves, or pathological Q waves, accompanied by symptoms such as chest tightness, palpitations, or chest pain.
(2) Time from symptom onset to emergency department presentation was no more than 12 h.

### Exclusion criteria

(1) Coagulation dysfunction or hereditary coagulation disorders; (2) Cardiogenic shock or severe heart failure (Killip class IV) at presentation; (3) Patient or family refusal to receive emergency percutaneous coronary intervention (PCI); (4) Comorbid conditions including Alzheimer's disease, renal insufficiency, or malignant tumor; (5) Pregnant or lactating women (17).

During this revision, the source clinical records were re-audited, and the group-specific AMI subtype (STEMI vs. NSTEMI), reperfusion modality [primary percutaneous coronary intervention (PCI) vs. thrombolysis], and disaggregated reperfusion-timing fields were recovered and verified. These variables are now reported in full: the AMI subtype distribution and reperfusion-modality utilization for each group are presented in Table 1, and the modality-specific reperfusion-timing metrics (door-to-balloon time among patients undergoing primary PCI and door-to-needle time among patients undergoing thrombolysis) are presented in Supplementary Table S3. The cohort was predominantly STEMI, consistent with the orientation of the procedural pathway, and the proportions of STEMI and of each reperfusion modality were comparable between groups. Because in-hospital mortality and MACE events were few, formal subtype-stratified outcome subgroup analyses remained statistically underpowered and are therefore reported descriptively rather than as definitive comparisons.

**Table 1:** Baseline demographic and clinical characteristics.

Characteristic	Intervention group (IG; *n* = 62)	Control group (CG; *n* = 64)	*p*-value
Age, years	56.30 ± 4.15	56.42 ± 4.38	0.875
Male sex, *n* (%)	36 (58.1)	38 (59.4)	0.881
Female sex, *n* (%)	26 (41.9)	26 (40.6)	
Body mass index, kg/m^2^	24.88 ± 2.20	24.90 ± 2.18	0.959
Symptom onset to emergency department presentation, h	2.03 ± 0.70	2.06 ± 0.58	0.794
Killip class II, *n* (%)	40 (64.5)	38 (59.4)	0.499
Killip class III, *n* (%)	22 (35.5)	26 (40.6)	
Coronary heart disease, *n* (%)	40 (64.5)	42 (65.6)	0.895
Hypertension, *n* (%)	22 (35.5)	31 (48.4)	0.142
Diabetes mellitus, *n* (%)	29 (46.8)	33 (51.6)	0.591
STEMI, *n* (%)	52 (83.9)	53 (82.8)	0.870
NSTEMI, *n* (%)	10 (16.1)	11 (17.2)	
Primary PCI, *n* (%)	40 (64.5)	42 (65.6)	0.895
Thrombolysis, *n* (%)	22 (35.5)	22 (34.4)	

Values are mean ± SD unless otherwise indicated. *p*-values for continuous variables were calculated using independent-samples *t*-tests; categorical variables were compared using the chi-square test or Fisher's exact test. Comorbidities are not mutually exclusive. All enrolled patients underwent reperfusion by either primary PCI or thrombolysis. Modality-specific door-to-balloon and door-to-needle times are reported in Supplementary Table S3. AMI, acute myocardial infarction; NSTEMI, non-ST-segment elevation myocardial infarction; PCI, percutaneous coronary intervention; SD, standard deviation; STEMI, ST-segment elevation myocardial infarction.

### Interventions

The cohort comprised patients with AMI managed through the emergency department. The intervention was particularly relevant to STEMI because the pathway emphasized rapid ECG recognition, early antiplatelet therapy, catheterization laboratory notification, and thrombolysis when timely PCI was not feasible. For patients with NSTEMI or suspected ischemia without persistent ST-segment elevation, the same graded triage and zoning workflow was applied, but invasive evaluation and reperfusion timing followed risk-based guideline recommendations rather than fixed STEMI timing windows. Patients in the CG received indication-based pharmacological and reperfusion therapies as clinically appropriate; however, their registration, triage, examination, and team activation followed routine sequential practice rather than a pre-specified, time-bound protocol.

### Control group: routine emergency nursing management

Patients in the CG received standard routine emergency nursing management. Nurses closely monitored vital signs, including breathing, heart rate, blood pressure, body temperature, and ECG changes. If symptoms such as breathing difficulties, confusion, or pallor were observed, immediate oxygen supplementation was provided, and the attending physician was notified. Pain management was administered according to physician orders using appropriate analgesics (e.g., morphine or pethidine), supplemented by psychological counseling and distraction techniques.

During the acute phase, the patients were maintained on absolute bed rest with fasting and avoidance of unnecessary activities. Following stabilization, nurses gradually guided patients through progressive activities, advancing from bed rest to bedside activity to indoor movement, and transitioned dietary intake from liquid to semi-liquid to regular food. Nursing staff also provided psychological support through communication and counseling to address the anxiety, fear, and despair commonly experienced by patients with AMI and educated patients and family members about the causes, symptoms, and treatment of AMI. Reperfusion (PCI or thrombolysis, as clinically indicated) was performed; however, the workflow was not pathway-driven, and pre-procedural steps (registration, triage, examination, and team activation) followed a standard sequential process rather than a pre-specified time-bound protocol.

### Intervention group: emergency procedural pathway combined with graded zoning management

**Figure 1:**
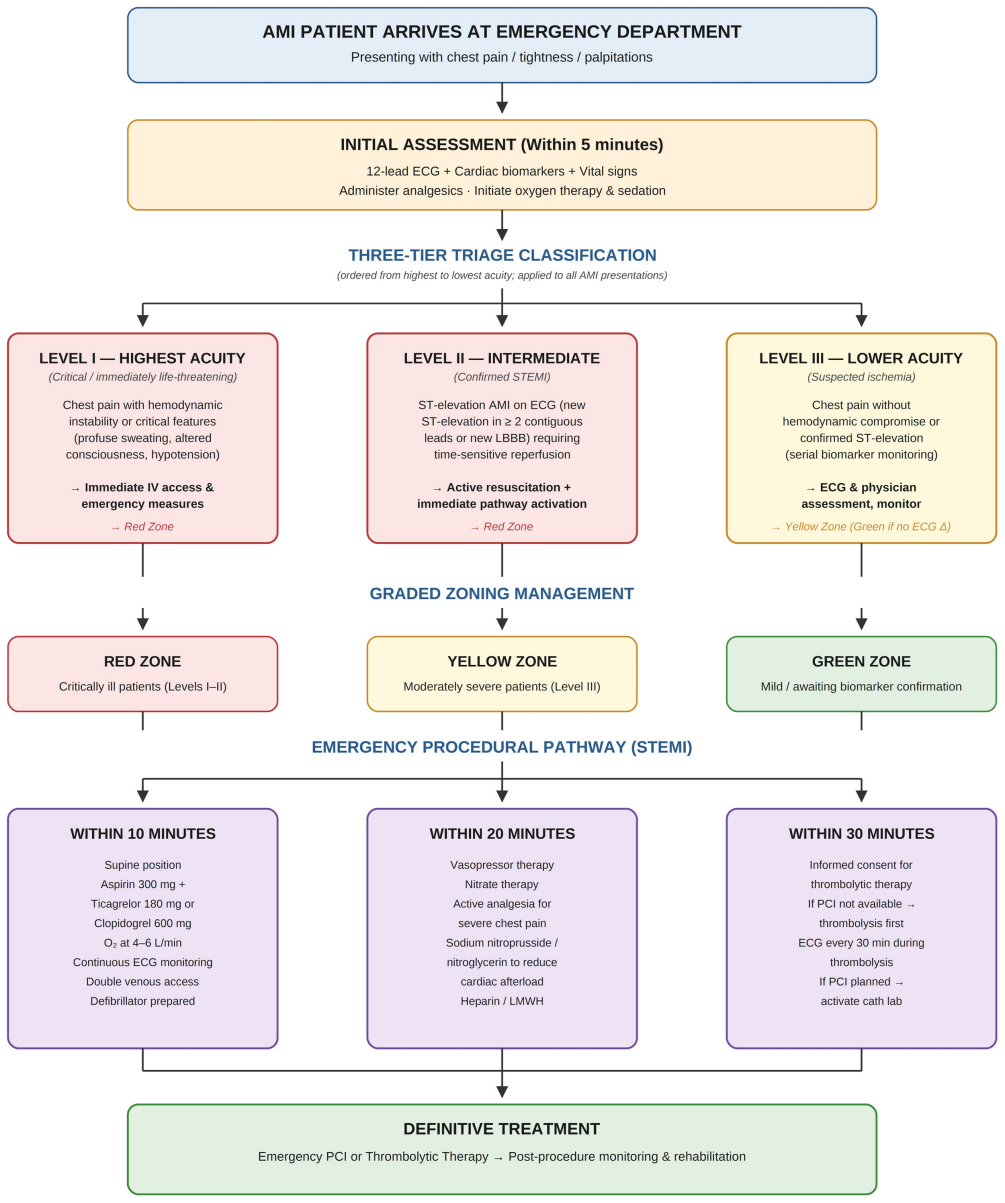
Schematic overview of the emergency procedural pathway combined with graded zoning management protocol. Upon arrival, patients underwent initial assessment within 5 min, followed by classification into a three-tier triage system (Level I, highest acuity: hemodynamic instability/critical features; Level II, confirmed STEMI requiring time-sensitive reperfusion; Level III, suspected ischemia without confirmed STEMI) and assignment to corresponding functional zones (Red, Yellow, or Green). The emergency procedural pathway comprised three timed phases for STEMI: initial stabilization and dual antiplatelet loading within 10 min, vasopressor and anticoagulation therapy within 20 min, and decision for thrombolytic therapy or catheterization laboratory activation within 30 min. For non-STEMI presentations, the same triage and zoning workflow applied, with reperfusion timing guided by risk-based recommendations rather than the STEMI-specific timing windows. AMI, acute myocardial infarction; ECG, electrocardiogram; PCI, percutaneous coronary intervention; LMWH, low-molecular-weight heparin; STEMI, ST-segment elevation myocardial infarction; NSTEMI, non-ST-segment elevation myocardial infarction.

Patients in the IG received emergency procedural pathway nursing combined with graded zoning management, which comprised two integrated components (Figure 1).

Construction of the emergency procedural pathway. A STEMI emergency team was established, consisting of emergency physicians, head nurses, and emergency department nurses, all of whom received professional training in cardiac care. Through collective discussion and evidence-based analysis, the team developed detailed nursing pathway plans for acute STEMI. For patients without persistent ST-segment elevation, the same triage and zoning structure was applied, and invasive assessment was guided by risk-based recommendations. Prior to implementation, nurses were trained to rapidly assess and classify patients using vital signs, ECG findings, cardiac enzymes, and other examinations to determine AMI status and severity and to formulate corresponding care plans.

Within 10 min of arrival at the emergency department, patients with acute STEMI were placed in a supine position and administered 300 mg of either aspirin with 180 mg of ticagrelor or 600 mg of clopidogrel under the guidance of emergency personnel. Oxygen inhalation at 4–6 L/min, continuous ECG monitoring, and double venous access were established, and a defibrillator was prepared for emergency use. Within 20 min, patients received vasopressor and nitrate therapy per physician orders. Patients with severe anterior chest pain received active analgesic treatment; sodium nitroprusside or nitroglycerin was used to reduce cardiac afterload, and heparin or low-molecular-weight heparin was administered to prevent thrombus expansion and vessel re-occlusion.

Within 30 min, the families of patients suitable for thrombolytic therapy were informed of the potential complications and necessary interventions. Patients who could not undergo emergency PCI in time received thrombolytic therapy after procedural (treatment) informed consent was obtained. During thrombolysis, the ECG was monitored every 30 min to observe coronary reperfusion. For patients planned for emergency PCI, preoperative preparations were completed, and the catheterization laboratory was activated.

#### Graded zoning management

Emergency personnel completed ECG and cardiac biomarker testing within 5 min for patients presenting with chest pain, administered oral or injectable analgesics, and initiated symptomatic treatment, including oxygen and sedation. Patients with confirmed ST-segment elevation were promptly transferred to the emergency resuscitation room, where experienced nurses rapidly established intravenous access and initiated rescue procedures.

Patient classification followed a three-tier triage system applied to all chest pain presentations consistent with possible AMI, with levels ordered from highest to lowest acuity:

- Level I, highest acuity (critical, immediately life-threatening): patients with chest pain accompanied by hemodynamic instability or other life-threatening features (e.g., profuse sweating, altered consciousness, and severe hypotension). These patients received immediate intravenous access, detailed vital sign monitoring, corresponding emergency measures, and were directed to the red zone.
- Level II, intermediate acuity (confirmed STEMI requiring time-sensitive reperfusion): patients with confirmed ST-segment elevation AMI on ECG (defined as new ST-segment elevation in two or more contiguous leads or new left bundle branch block); these patients received active resuscitation, immediate procedural pathway activation (cardiology consultation and catheterization laboratory notification), and assignment to the red zone.
- Level III, lower acuity (suspected ischemia without confirmed STEMI): patients with chest pain but without hemodynamic compromise or confirmed ST-segment elevation; triage nurses performed ECG and prompt physician assessment, with continued monitoring of myocardial biomarker levels. These patients were assigned to the yellow zone, with reassignment to the red zone if the criteria for Level I or II subsequently developed. Patients without ECG abnormalities were managed in the green zone with serial myocardial marker monitoring.

This three-tier hierarchy was designed to ensure that the most clinically unstable patients (Level I) received the fastest response, while confirmed STEMI patients (Level II) received a parallel rapid activation of the reperfusion pathway. The system was applied to the full AMI cohort, not only to STEMI presentations.

The emergency department was divided into three functional zones: a red zone for critically ill patients (Levels I and II), a yellow zone for patients with moderately severe conditions (Level III), and a green zone for patients with relatively mild conditions or those awaiting biomarker confirmation. During treatment, nurses communicated promptly with senior medical staff and reserved limited resources for the most severely ill patients. The registration, triage, and examination processes were simplified to minimize delays.

### Outcome measures

The primary outcome was in-hospital survival to discharge, defined as the proportion of patients alive at hospital discharge following index AMI admission. Survival status was determined from the medical records at the time of discharge by the attending team and verified by an independent emergency department clinician not involved in the care of the patient. The secondary outcomes included first-aid time, length of hospital stay, incidence of major adverse cardiovascular events (MACE), quality of life, psychological status, and patient satisfaction.

First-aid efficiency. First-aid time was defined as the interval, in minutes, from emergency department arrival to the initiation of definitive reperfusion treatment, defined as the first thrombolytic bolus for fibrinolysis or arrival in the catheterization laboratory for primary PCI. This local composite metric was retained because it was consistently available for every patient in the study database. It does not correspond exactly to internationally standardized reperfusion indicators such as door-to-balloon time, door-to-needle time, or first-medical-contact-to-device time. To address this point, the standardized, modality-specific reperfusion intervals were recovered during revision and are now reported separately: door-to-balloon time for patients undergoing primary PCI and door-to-needle time for patients undergoing thrombolysis are summarized in the Results and presented in full in Supplementary Table S3. The length of hospital stay was recorded as the total number of days from admission to discharge.

#### Major adverse cardiovascular events

The incidence of MACE was recorded during the index hospitalization. MACE in this study comprised heart failure (newly developed or decompensated, defined by clinical signs plus echocardiographic confirmation), recurrent myocardial infarction (defined by recurrent ischemic symptoms with new ECG changes and/or re-elevation of troponin), clinically significant arrhythmia (defined as ventricular tachycardia, ventricular fibrillation, sustained supraventricular tachycardia requiring intervention, or new high-grade atrioventricular block; isolated, asymptomatic ectopy was not counted), and cardiogenic shock. In-hospital all-cause death was captured separately under the primary outcome (in-hospital survival) and was therefore not double-counted within the MACE composite. Adverse events were extracted from the hospital electronic medical record by a research nurse and adjudicated by an emergency physician not directly involved in the patient's care; disagreements were resolved by consensus discussion with a senior cardiology consultant.

#### Quality of life

Quality of life was assessed using the Generic Quality of Life Inventory-74 (GQOL-74) (18), which evaluates four domains: physical, social, psychological, and material life status. Each domain yields a maximum score of 100, with higher scores indicating a better quality of life. The GQOL-74 was administered upon discharge. The Cronbach's α coefficient of this scale in the present study was 0.867, indicating a good internal consistency. The GQOL-74 is a generic instrument; its validation in AMI populations specifically remains limited (7, 19), and findings should therefore be interpreted with this caveat. A baseline pre-intervention QoL assessment was not feasible because of the acute and time-pressured nature of the presentation.

#### Psychological status

Psychological status was assessed using the Self-Rating Anxiety Scale (SAS) (20) and the Self-Rating Depression Scale (SDS) (21) both before and after the intervention. The total scores for each scale range from 25 to 100; an SAS score ≥ 50 indicates anxiety symptoms, and an SDS score ≥ 53 indicates depressive symptoms, with lower scores reflecting better psychological wellbeing. Baseline assessments were conducted within 24 h of admission following initial stabilization, and post-intervention assessments were performed at the time of discharge. The Cronbach's α coefficients in this study were 0.857 for the SAS and 0.856 for the SDS.

#### Patient satisfaction

Patient satisfaction was assessed at discharge using the Newcastle Nursing Satisfaction Scale (NSNS) (22). Scores of 95, 76–94, 57–75, 38–56, and 19–37 represented “very satisfied,” “satisfied,” “generally satisfied,” “dissatisfied,” and “very dissatisfied,” respectively. Total satisfaction was defined as the proportion of patients scoring ≥ 57 (“generally satisfied” or above). Cronbach's α coefficient was 0.885.

Owing to the nature of the intervention, blinding of clinical staff and patients was not feasible. This creates a risk of performance bias because staff awareness of the new pathway could reasonably lead to greater vigilance, faster communication, and closer follow-up beyond the formal pathway elements. To reduce ascertainment bias, the outcome assessors responsible for administering the GQOL-74, SAS, SDS, and NSNS questionnaires were blinded to group allocation. MACE adjudicators were also blinded to group allocation.

### Statistical analysis

Data analysis was performed using SPSS version 26.0 (IBM Corp., Armonk, NY, USA). Continuous variables were assessed for normality using the Shapiro–Wilk test. Normally distributed continuous data are presented as mean ± standard deviation (SD) and were compared between groups using independent-samples *t*-tests; within-group pre–post comparisons were performed using paired-samples *t*-tests. All continuous outcomes in this study met the assumption of normality; therefore, non-parametric alternatives were not required. Categorical data are expressed as n (%) and were compared using the chi-square test or Fisher's exact test when expected cell frequencies were less than 5.

For continuous outcomes, mean differences with 95% confidence intervals (CIs) were reported. For categorical outcomes, risk differences with 95% CIs (Wald method) were reported, and odds ratios (ORs) with 95% CIs were calculated where appropriate.

To address the non-randomized, sequential-enrollment design and the possibility of residual baseline imbalance, pre-specified multivariable analyses were performed for the primary outcome (in-hospital survival to discharge) and for the MACE composite using logistic regression adjusted for age, sex, BMI, Killip class, and principal comorbidities (coronary heart disease, hypertension, diabetes mellitus). Because in-hospital deaths (n = 14) and MACE (n = 16) were few, the number of events per candidate covariate was low; the adjusted models were therefore deliberately restricted to a small, pre-specified set of confounders to limit overfitting, and the resulting wide confidence intervals reflect this statistical fragility. Because mortality and MACE events were relatively few, the adjusted models were intentionally parsimonious and should be considered exploratory. Infarct territory, reperfusion eligibility, and hemodynamic instability beyond Killip classification were not available in a complete audit-ready format for inclusion in the models; reperfusion modality, although now reported descriptively (Table 1, Supplementary Table S3), was likewise not entered into the adjustment models, because adding further covariates to the small number of events would have aggravated overfitting. Adjusted odds ratios (aORs) with 95% CIs are reported.

The primary outcome was pre-specified, and findings for secondary outcomes were interpreted as exploratory; no formal correction for multiplicity was applied to secondary endpoints. The significance threshold was set at a two-sided *p* < 0.05.

## Results

### Baseline characteristics and group comparability

**Figure 2:**
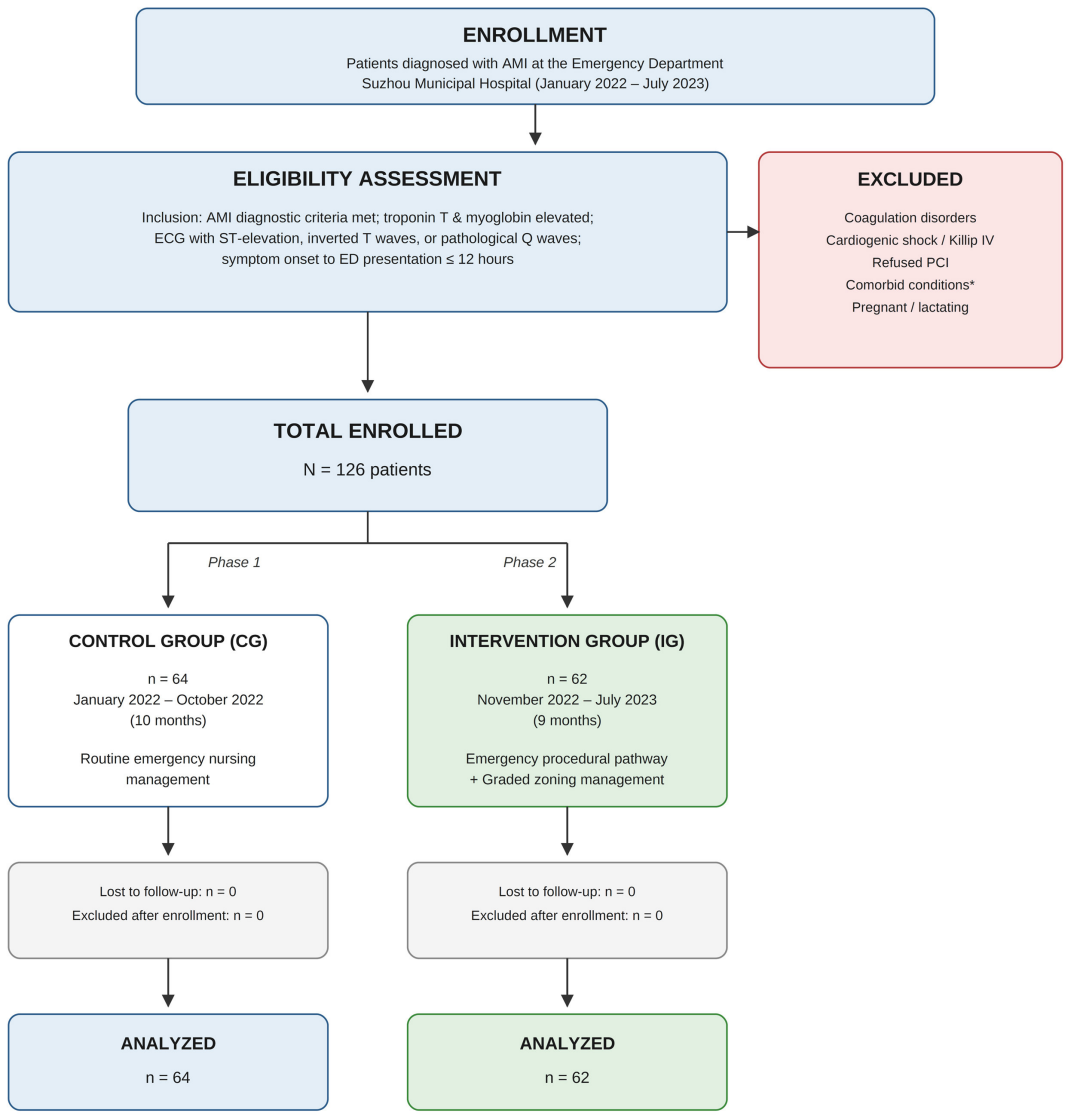
Patient flow diagram. A total of 126 patients diagnosed with acute myocardial infarction were enrolled using a sequential pre–post implementation design. Patients admitted during Phase 1 (January 2022–October 2022) received routine emergency nursing and constituted the control group (CG; *n* = 64), while patients admitted during Phase 2 (November 2022–July 2023) received emergency procedural pathway combined with graded zoning management and constituted the intervention group (IG; *n* = 62). No patients were lost to follow-up or excluded after enrollment. *Comorbid conditions: Alzheimer's disease, renal insufficiency, or malignant tumor. AMI, acute myocardial infarction; PCI, percutaneous coronary intervention.

Of the 126 patients enrolled, 62 were allocated to the intervention group (IG) and 64 to the control group (CG). No patients were lost to follow-up or excluded after enrollment; thus, all 126 patients were included in the final analysis (Figure 2). The baseline demographic and clinical characteristics of the two groups are summarized in Table 1. The IG comprised 36 males and 26 females with a mean age of 56.30 ± 4.15 years, while the CG comprised 38 males and 26 females with a mean age of 56.42 ± 4.38 years. Body mass index, onset-to-presentation time, Killip class, the principal comorbidities (coronary heart disease, hypertension, and diabetes mellitus), the distribution of AMI subtype (STEMI vs. NSTEMI), and reperfusion-modality utilization (primary PCI vs. thrombolysis) did not differ significantly between groups (all *p* > 0.05; Table 1). The cohort was predominantly STEMI in both groups (83.9% in the IG and 82.8% in the CG). These observed similarities support between-group comparison for the measured variables but do not eliminate residual confounding from unmeasured AMI severity, infarct territory, reperfusion eligibility, or temporal differences between enrollment periods.

### AMI subtype, reperfusion modality, and timing variables

During revision, the source clinical records were re-audited so that AMI subtype, reperfusion modality, and disaggregated reperfusion-timing metrics could be reported by group (Table 1; Supplementary Table S3). The cohort was predominantly STEMI, with 52 of 62 patients (83.9%) in the IG and 53 of 64 patients (82.8%) in the CG classified as STEMI; the remaining patients had NSTEMI [10 [16.1%] in the IG and 11 [17.2%] in the CG]. The proportion of STEMI did not differ significantly between groups (*p* = 0.870). All enrolled patients underwent reperfusion. Primary PCI was performed in 40 patients (64.5%) in the IG and 42 patients (65.6%) in the CG, whereas thrombolysis was used in 22 patients (35.5%) in the IG and 22 patients (34.4%) in the CG; reperfusion-modality utilization was comparable between groups (*p* = 0.895). Standardized reperfusion-timing metrics favored the IG across both modalities (Supplementary Table S3). Among patients undergoing primary PCI, door-to-balloon time was shorter in the IG than in the CG (78.5 ± 12.3 vs. 92.4 ± 15.6 min; mean difference −13.9 min, 95% CI −20.0 to −7.8; *p* < 0.001). Among patients undergoing thrombolysis, door-to-needle time was likewise shorter in the IG (32.5 ± 6.8 vs. 41.2 ± 8.9 min; mean difference −8.7 min, 95% CI −13.5 to −3.9; *p* < 0.001). Because the numbers of in-hospital deaths and MACE were small, formal subtype-stratified outcome subgroup analyses were statistically underpowered; the directional consistency of the timing improvements within both the STEMI-predominant PCI and thrombolysis subgroups is therefore presented descriptively and should be interpreted with caution.

### Primary outcome: in-hospital survival to discharge

In-hospital survival to discharge was higher in the IG than in the CG (96.8%, 60/62 vs. 81.3%, 52/64; risk difference 15.5%, 95% CI 4.7% to 26.4%; Fisher's exact *p* = 0.008; Table 2). After multivariable adjustment for age, sex, BMI, Killip class, and comorbidities (coronary heart disease, hypertension, and diabetes mellitus), the association remained statistically significant (adjusted OR 6.50, 95% CI 1.32 to 32.0, *p* = 0.022). Two patients in the IG and 12 patients in the CG died during the index hospitalization, corresponding to in-hospital mortality rates of 3.2% and 18.7%, respectively. Given the wide confidence interval and small number of deaths, this adjusted estimate should be interpreted cautiously.

**Table 2:** Clinical, reperfusion-timing, in-hospital adverse-event, and patient-reported outcomes.

Outcome	Intervention group (IG; *n* = 62)	Control group (CG; *n* = 64)	Between-group estimate (95% CI)	*p*-value
First-aid time, min	67.30 ± 3.90	75.70 ± 4.50	Mean difference −8.40 (−9.87 to −6.93)	< 0.001
Length of hospital stay, days	14.62 ± 2.05	18.08 ± 4.55	Mean difference −3.46 (−4.69 to −2.23)	< 0.001
In-hospital survival to discharge, *n* (%)	60 (96.8)	52 (81.3)	Risk difference +15.5% (4.7%−26.4%); adjusted OR 6.50 (1.32–32.0)	0.008; adjusted *p* = 0.022
In-hospital mortality, *n* (%)	2 (3.2)	12 (18.7)	Risk difference −15.5% (−26.4% to −4.7%)	0.008
Total MACE, *n* (%)	4 (6.5)	12 (18.8)	Risk difference −12.3% (−23.0% to −1.6%); adjusted OR 0.30 (0.09–0.97)	0.042; adjusted *p* = 0.045
Heart failure, *n* (%)	2 (3.2)	5 (7.8)	Descriptive component of MACE	
Recurrent MI, *n* (%)	1 (1.6)	3 (4.7)	Descriptive component of MACE	
Clinically significant arrhythmia, *n* (%)	1 (1.6)	2 (3.1)	Descriptive component of MACE	
Cardiogenic shock, *n* (%)	0 (0.0)	2 (3.1)	Descriptive component of MACE	
Door-to-balloon time, min (primary PCI)	78.5 ± 12.3	92.4 ± 15.6	Mean difference −13.9 (−20.0 to −7.8)	< 0.001
Door-to-needle time, min (thrombolysis)	32.5 ± 6.8	41.2 ± 8.9	Mean difference −8.7 (−13.5 to −3.9)	< 0.001
GQOL-74 physical function at discharge	72.45 ± 4.80	65.30 ± 4.25	Mean difference 7.15 (5.56–8.74)	< 0.001
GQOL-74 social function at discharge	73.68 ± 6.50	62.14 ± 5.90	Mean difference 11.54 (9.37 to 13.71)	< 0.001
GQOL-74 psychological function at discharge	68.52 ± 5.85	55.20 ± 4.65	Mean difference 13.32 (11.47–15.17)	< 0.001
GQOL-74 material life status at discharge	64.38 ± 9.10	57.42 ± 8.75	Mean difference 6.96 (3.84–10.08)	< 0.001
SAS before intervention	46.12 ± 5.00	46.08 ± 5.10	Mean difference 0.04	0.964
SAS after intervention	31.70 ± 6.85	38.05 ± 7.24	Mean difference −6.35 (−8.81 to −3.89)	< 0.001
SDS before intervention	41.22 ± 2.98	41.30 ± 3.25	Mean difference −0.08	0.883
SDS after intervention	34.10 ± 3.20	36.28 ± 3.18	Mean difference −2.18 (−3.30 to −1.06)	< 0.001
Total satisfaction at discharge, *n* (%)	54 (87.1)	42 (65.6)	Risk difference +21.5% (7.4%−35.6%)	0.005

Mean difference values are IG minus CG. Fisher's exact test was used for in-hospital survival and total MACE. Adjusted odds ratios were estimated using parsimonious logistic regression adjusted for age, sex, BMI, Killip class, and principal comorbidities. Because event counts were small and confidence intervals were wide, adjusted estimates should be interpreted as exploratory. MACE comprised heart failure, recurrent MI, clinically significant arrhythmia, and cardiogenic shock; in-hospital death was analyzed separately and was not double-counted within the MACE composite. Door-to-balloon and door-to-needle times are reported among patients receiving primary PCI and thrombolysis, respectively (see also Supplementary Table S3). GQOL-74 scores range from 0 to 100 by domain, with higher scores indicating better quality of life; the GQOL-74 was administered at discharge, and no baseline quality-of-life measurement was available. SAS and SDS scores are lower when psychological status is better (clinical thresholds SAS ≥ 50 and SDS ≥ 53); pre-intervention assessments were obtained within 24 h of admission and post-intervention assessments at discharge. Satisfaction was measured with the Newcastle Nursing Satisfaction Scale, and total satisfaction was defined as “generally satisfied” or above. CI, confidence interval; MACE, major adverse cardiovascular events; MI, myocardial infarction; OR, odds ratio; GQOL-74, Generic Quality of Life Inventory-74; PCI, percutaneous coronary intervention; SAS, Self-rating Anxiety Scale; SDS, Self-rating Depression Scale.

### First-aid efficiency and length of hospital stay

The implementation of the emergency procedural pathway combined with graded zoning management was associated with improved first-aid efficiency (Table 2). Patients in the IG demonstrated a shorter mean first-aid time compared with the CG (67.30 ± 3.90 min vs. 75.70 ± 4.50 min; mean difference −8.40 min, 95% CI −9.87 to −6.93; t = 11.21, *p* < 0.001), representing a mean reduction of approximately 8.4 min in the locally defined door-to-treatment interval. The mean length of hospital stay was shorter in the IG than in the CG (14.62 ± 2.05 days vs. 18.08 ± 4.55 days; mean difference −3.46 days, 95% CI −4.69 to −2.23; *t* = 5.54, *p* < 0.001).

### Incidence of major adverse cardiovascular events

The overall incidence of MACE during index hospitalization was lower in the IG than in the CG (6.5%, 4/62 vs. 18.8%, 12/64; risk difference −12.3%, 95% CI −23.0% to −1.6%; Fisher's exact *p* = 0.042; Table 2). After multivariable adjustment, the association remained statistically significant but imprecise (adjusted OR 0.30, 95% CI 0.09 to 0.97, *p* = 0.045). In the IG, adverse events included heart failure in two patients (3.2%), recurrent myocardial infarction in one patient (1.6%), and clinically significant arrhythmia in one patient (1.6%), and no cases of cardiogenic shock were recorded. In contrast, the CG experienced heart failure in five patients (7.8%), recurrent myocardial infarction in three (4.7%), arrhythmia in two (3.1%), and cardiogenic shock in two (3.1%).

### Quality of life

Quality of life at discharge, assessed using the GQOL-74, was higher in the IG across all four domains (Table 2). Physical function, social function, psychological function, and material life status scores all favored the IG, with the largest between-group difference in the psychological function domain. These findings indicate that the combined intervention was associated with better patient-reported quality of life at discharge; however, because no baseline QoL measurement was obtained, these between-group differences cannot be interpreted as within-patient changes.

### Psychological status

Baseline SAS and SDS scores were comparable between the two groups at admission, confirming the absence of measured pre-intervention differences in anxiety and depression scores (SAS: IG 46.12 ± 5.00 vs. CG 46.08 ± 5.10, *p* = 0.964; SDS: IG 41.22 ± 2.98 vs. CG 41.30 ± 3.25, *p* = 0.883; Table 2). Following the intervention, both groups exhibited statistically significant within-group reductions in anxiety and depression scores, indicating that both nursing approaches were associated with psychological improvement during hospitalization.

The magnitude of improvement was greater in the IG. Post-intervention SAS scores were lower in the IG than in the CG (31.70 ± 6.85 vs. 38.05 ± 7.24; mean difference −6.35, 95% CI −8.81 to −3.89; *t* = 5.06, *p* < 0.001), and post-intervention SDS scores similarly favored the IG (34.10 ± 3.20 vs. 36.28 ± 3.18; mean difference −2.18, 95% CI −3.30 to −1.06; *t* = 3.83, *p* < 0.001). Notably, the post-intervention mean scores in both groups remained below the clinical thresholds for anxiety (SAS ≥ 50) and depression (SDS ≥ 53).

### Patient satisfaction

Patient satisfaction, assessed at discharge using the NSNS, was higher in the IG (Table 2). The total satisfaction rate, defined as the proportion of patients scoring “generally satisfied” or above (≥ 57), reached 87.1% (54/62) in the IG compared with 65.6% (42/64) in the CG (risk difference 21.5%, 95% CI 7.4% to 35.6%; χ^2^ = 8.00, *p* = 0.005). These findings suggest that the structured, time-sensitive approach of the combined intervention was associated with an enhanced patient experience of emergency cardiac care.

## Discussion

The principal finding of this study is that implementation of an emergency procedural pathway combined with graded zoning management was associated with higher in-hospital survival to discharge, shorter first-aid time, shorter hospital stay, lower in-hospital MACE, and better discharge patient-reported outcomes among patients with AMI. These findings suggest that a structured emergency nursing workflow may improve the coordination and timeliness of AMI care in a high-volume emergency department. However, the magnitude of the mortality difference was large for a workflow intervention, and the non-randomized sequential design prevents causal attribution. The results should therefore be interpreted as hypothesis-generating associations requiring confirmation in rigorously designed multicenter studies.

These results are consistent with prior evidence that time-sensitive emergency pathways can improve key process measures in STEMI and acute coronary syndrome care (13, 23). The mechanistic plausibility of our findings is supported by literature showing that earlier ECG acquisition and shorter emergency department delays are associated with better outcomes in STEMI care (9, 23). Söner et al. reported that emergency department delay time independently predicted 1-year all-cause mortality after primary PCI in STEMI patients, underscoring the importance of reducing early treatment delays (9). In addition, incomplete ST-segment resolution after primary PCI has been linked to adverse clinical outcomes, suggesting that timely care is necessary but not sufficient, and that infarct characteristics and reperfusion effectiveness remain important determinants of prognosis (8). Quantitatively, our own data are concordant with these reports: the procedural pathway was associated with a reduction of approximately 13.9 min in door-to-balloon time among patients undergoing primary PCI and approximately 8.7 min in door-to-needle time among those undergoing thrombolysis (Supplementary Table S3). Because each minute of reperfusion delay has been linked to incremental mortality risk after primary PCI (9), reductions of this magnitude are clinically plausible mediators of the better outcomes observed in the intervention group. Nevertheless, the absolute survival difference observed here is considerably larger than would be predicted from the reperfusion-timing improvements alone. Published continuous dose-response estimates indicate that each 30-minute reduction in time to reperfusion is associated with roughly a 7.5% relative decrease in subsequent mortality (24); although these estimates derive principally from 1-year outcomes in primary-PCI cohorts and therefore furnish only an approximate benchmark for in-hospital survival, their order of magnitude is instructive. Applied to the baseline mortality of the present cohort, reductions of approximately 13.9 min in door-to-balloon time and 8.7 min in door-to-needle time would be expected to translate into an absolute mortality benefit of, at most, roughly one to two percentage points, rather than the 15.5-percentage-point absolute survival difference recorded between the groups. This discrepancy indicates that the faster reperfusion intervals account for only part of the observed effect, and that additional factors, most plausibly unmeasured differences in AMI severity, infarct territory, and reperfusion eligibility, together with the secular and case-mix changes inherent to the sequential pre-post design, contributed materially to the mortality gap. The timing data are accordingly best read as evidence that the pathway performed as intended in compressing treatment delay, rather than as a sufficient mechanistic explanation for the full magnitude of the survival difference, which we therefore continue to interpret as an association rather than a causal effect. The direction and approximate magnitude of our findings also align with earlier reports that optimization of emergency nursing workflows before coronary intervention shortens treatment delays and improves outcomes in AMI (14), and that structured emergency nursing and monitoring procedures are associated with improved recovery in other acute cardiovascular and cerebrovascular conditions (25). Taken together with the broader pathophysiology of myocardial infarction, in which prolonged ischemia drives progressive myocyte loss and mechanical complications (26–28), these convergent lines of evidence support the biological rationale for minimizing avoidable in-hospital delay.

The in-hospital mortality of 18.7% observed in the control group also merits comment, because it exceeds the 4% to 6% commonly reported in contemporary STEMI registries, notwithstanding the relatively young mean age of the cohort and the exclusion of patients in Killip class IV or with cardiogenic shock at presentation, severe comorbidities, or refusal of percutaneous coronary intervention. Several considerations may account for this rate. The cohort still included a sizeable proportion of patients in Killip classes II and III, in whom acute heart failure carries a non-trivial in-hospital mortality. The routine-care era was characterized by non-protocolized, sequential triage with longer door-to-treatment times in a high-volume emergency department, which may itself have elevated early mortality. The control- group estimate, moreover, rests on only 12 deaths among 64 patients, so the point estimate is statistically imprecise and its confidence interval correspondingly wide; unmeasured baseline severity together with the secular differences between enrollment periods may have further inflated the control-group rate relative to the intervention period. Taken together, these considerations reinforce that the between-group mortality difference should be regarded as an association attended by meaningful residual uncertainty rather than as a precise estimate of intervention benefit.

The observed reduction in MACE and the higher quality-of-life scores in the IG may reflect several interacting mechanisms. The graded classification system supported rapid identification of clinically unstable patients and patients with ECG evidence of STEMI, while zoning facilitated prioritization of beds, nurses, monitoring capacity, and physician response. The procedural pathway reduced unnecessary sequential steps by simplifying registration, triage, examination, and team activation. These changes may have contributed to earlier treatment and more consistent monitoring, plausibly limiting the ischemic burden that precipitates heart failure, malignant arrhythmia, and mechanical complications after myocardial infarction (28). Nevertheless, although AMI subtype, reperfusion modality, and modality-specific reperfusion timing are now reported (Table 1; Supplementary Table S3), the study still lacked complete infarct-territory, culprit-vessel, and reperfusion-eligibility data; consequently, the exact contribution of workflow redesign relative to underlying AMI severity cannot be fully disentangled.

The better post-intervention SAS, SDS, and satisfaction scores in the IG may also be explained by the structure and predictability of care. Standardized nursing pathways can reduce uncertainty for patients and family members by clarifying priorities, expediting communication, and providing visible continuity in monitoring and treatment. This interpretation is consistent with evidence that structured emergency department nursing interventions can favorably influence psychological symptoms and self-care capacity in acutely ill patients (29). These effects may improve the patient experience even when clinical severity remains substantial. Because these patient-reported outcomes were measured at discharge, however, they should be interpreted as short-term perceptions of recovery and care rather than durable long-term improvements. More fundamentally, because both patients and treating staff were necessarily aware of the new pathway, the favorable patient-reported outcomes are especially susceptible to expectation and performance effects. Heightened staff engagement, the visible novelty of a structured pathway, and the patients' own awareness that they were receiving an enhanced model of care may have elevated the reported quality-of-life and satisfaction scores, and lowered the reported anxiety and depression scores, independently of any true difference in experience. Blinding the assessors who administered the GQOL-74, SAS, SDS, and NSNS instruments mitigates observer bias, but it cannot eliminate this expectation effect, because the patient remained the source of each rating. The patient-reported findings should therefore be regarded as the most provisional of the study's outcomes and weighed accordingly.

This study has several limitations that should be acknowledged. First, this was a single-center study conducted at one hospital with a relatively modest sample size (*n* = 126), which may limit the generalizability of the findings to other settings, populations, and healthcare systems. Second, the quasi-experimental design with sequential pre-post enrollment, rather than concurrent randomization, introduced a substantial risk of temporal and secular confounding. Changes over time in staffing, operator experience, equipment, ancillary protocols, seasonal patient mix, hospital crowding, and learning effects may have influenced the outcomes independently of the intervention. The large observed mortality difference is therefore not attributable to the workflow intervention alone with certainty. Although the two enrollment phases were of comparable duration and multivariable adjustment was performed, residual confounding from unmeasured time-varying factors cannot be excluded. Accordingly, the observed differences should be interpreted as associations rather than definitive causal effects of the intervention.

Third, the adjustment model remained incomplete. Although AMI subtype and reperfusion modality are now reported and were shown to be balanced between groups (Table 1), important AMI severity variables, including infarct territory, culprit-vessel distribution, reperfusion eligibility, and hemodynamic instability beyond Killip classification, were not available in complete audit-ready form for modeling, and reperfusion modality itself was not entered into the regression in order to preserve a low number of covariates relative to events. These factors may materially influence mortality, MACE, and treatment timing, and their omission is particularly important because the intervention was STEMI-oriented; even modest residual imbalance in unmeasured severity could affect outcomes.

Fourth, group-specific STEMI/NSTEMI proportions, PCI vs. thrombolysis utilization rates, and door-to-balloon and door-to-needle times have now been recovered and are reported (Table 1; Supplementary Table S3). However, because the numbers of in-hospital deaths and MACE were small, formal AMI subtype-stratified outcome subgroup analyses were statistically underpowered and are presented descriptively rather than as confirmatory comparisons. Future studies should prospectively capture subtype, reperfusion strategy, infarct territory, and standardized reperfusion intervals in sufficiently large cohorts to permit adequately powered subgroup analyses.

Fifth, the regression analyses may be statistically unstable because of the small number of mortality and MACE events. The wide confidence intervals around the adjusted odds ratios indicate imprecision and possible overfitting despite the parsimonious model. The adjusted analyses should therefore be viewed as supportive exploratory analyses rather than definitive estimates of treatment effect.

Sixth, the intervention was delivered as a complex bundle that combined the emergency procedural pathway with graded zoning management, structured training, and teamwork. Because these components were implemented simultaneously, their individual contributions to the observed effects cannot be disentangled in this study, and improvements may also reflect non-specific effects of process standardization, heightened awareness, and team engagement.

Seventh, the lack of randomization and the unblinded nature of the intervention introduced the potential for selection bias and performance bias. Healthcare professionals could not be blinded, and awareness of the intervention may have led to closer attention or more proactive care in the IG. Outcome assessors and MACE adjudicators were blinded to group allocation to reduce ascertainment bias, but residual measurement and performance bias cannot be excluded. Because they depend on patient perception, the patient-reported outcomes (quality of life, satisfaction, anxiety, and depression) are the endpoints most exposed to this expectation and performance bias; blinded administration of the instruments addresses observer bias but not the patient's awareness of the model of care received, and the favorable patient-reported findings should be interpreted with this constraint firmly in mind.

Eighth, the exclusion of patients with cardiogenic shock or Killip class IV at presentation, those refusing PCI, and those with major comorbidities such as renal insufficiency, malignancy, or Alzheimer's disease limits external validity. The findings therefore reflect a relatively selected AMI cohort and may not apply to the highest-risk real-world AMI populations. In addition, the primary outcome was limited to survival to discharge and does not capture 30-day, 6-month, or 1-year mortality or MACE. Quality-of-life and satisfaction outcomes were assessed only at discharge, without a baseline QoL measure or long-term follow-up, and the GQOL-74 is a generic instrument with limited AMI-specific validation.

Future multicenter randomized or stepped-wedge cluster trials with concurrent controls, standardized AMI subtype documentation, reperfusion modality capture, door-to-balloon and door-to-needle metrics, infarct territory data, blinded endpoint adjudication, and longer follow-up are warranted to confirm and extend these preliminary findings.

## Conclusion

In this single-center quasi-experimental study, an emergency procedural pathway combined with graded zoning management was associated with improved in-hospital survival, first-aid efficiency, fewer in-hospital MACE, and better discharge quality-of-life and satisfaction scores in patients with AMI. Because the design was non-randomized and sequential, these findings should be interpreted as associations rather than causal effects and require confirmation in multicenter randomized or stepped-wedge trials before broad implementation.

## Data Availability

The raw data supporting the conclusions of this article will be made available by the authors, without undue reservation.

## References

[1] Pollard TJ. The acute myocardial infarction. Prim Care. (2000) 27:631–49. doi: 10.1016/S0095-4543(05)70167-6 10918673

[2] Dauerman HL Ibanez B. The edge of time in acute myocardial infarction. J Am Coll Cardiol. (2021) 77:1871–4. doi: 10.1016/j.jacc.2021.03.003 33858623

[3] Sandoval Y Jaffe AS. Type 2 myocardial infarction: JACC review topic of the week. J Am Coll Cardiol. (2019) 73:1846–60. doi: 10.1016/j.jacc.2019.02.018 30975302

[4] Wong CK White HD. Has the mortality rate from acute myocardial infarction fallen substantially in recent years? Eur Heart J. (2002) 23:689–92. doi: 10.1053/euhj.2001.3054 11977991

[5] Freestanding emergency departments. Ann Emerg Med. (2020) 76:e89–90. doi: 10.1016/j.annemergmed.2020.06.005 33012403

[6] Butt TS Bashtawi E Bououn B Wagley B Albarrak B Sergani HE . Door-to-balloon time in the treatment of ST segment elevation myocardial infarction in a tertiary care center in Saudi Arabia. Ann Saudi Med. (2020) 40:281–9. doi: 10.5144/0256-4947.2020.281 32757982 PMC7410222

[7] Dziewierz A Zdzierak B Wańha W Luca G Rakowski T. The irreversible march of time: ischemic delay and impact on outcomes in st-segment elevation myocardial infarction. J Cardiovasc Dev Dis. (2025) 12:474. doi: 10.3390/jcdd12120474 41440853 PMC12733498

[8] Söner S Özbek M. Predictors of ST-segment resolution and its relationship with outcomes in patients with ST-elevation myocardial infarction undergoing primary percutaneous coronary intervention. Angiology. (2025) 76:1010–8. doi: 10.1177/00033197251372771 40958745

[9] Söner S Taştan E Okşul M Cömert AD Inci Ü Öztürk C . The effect of emergency department delay time on all-cause mortality of ST-segment elevation myocardial infarction patients who underwent primary percutaneous coronary intervention: does every minute affect mortality? Adv Int Cardiol. (2025) 21:37–44. doi: 10.5114/aic.2024.145173 40182087 PMC11963035

[10] Bradley EH Herrin J Wang Y Barton BA Webster TR Mattera JA . Strategies for reducing the door-to-balloon time in acute myocardial infarction. N Engl J Med. (2006) 355:2308–20. doi: 10.1056/NEJMsa063117 17101617

[11] Jollis JG. Implementation of a statewide system for coronary reperfusion for ST-segment elevation myocardial infarction. JAMA. (2007) 298:2371. doi: 10.1001/jama.298.20.joc70124 17982184

[12] Terkelsen CJ. System delay and mortality among patients with STEMI treated with primary percutaneous coronary intervention. JAMA. (2010) 304:763. doi: 10.1001/jama.2010.1139 20716739

[13] Xiong W Gao W Zhou Q Wu Z. Clinical impact of an emergency fast-track pathway on early intervention and outcomes in acute coronary syndrome: a prospective cohort study. Sci Rep. (2025) 15:41509. doi: 10.1038/s41598-025-25333-5 41285841 PMC12644491

[14] Deng Y Song J. Application effect evaluation of optimizing emergency nursing process before emergency coronary intervention in patients with AMI and hypertension. Altern Ther Health Med. (2024) 30:339–43. 37820661

[15] Stengaard C Sørensen JT Rasmussen MB Bøtker MT Pedersen CK Terkelsen CJ. Prehospital diagnosis of patients with acute myocardial infarction. Diagnosis. (2016) 3:155–66. doi: 10.1515/dx-2016-0021 29536903

[16] Byrne R Coughlan JJ Rossello X Ibanez B. The ‘10 commandments' for the (2023). ESC guidelines for the management of acute coronary syndromes. Eur Heart J. (2024) 45:1193–95. doi: 10.1093/eurheartj/ehad863 38206306

[17] Zeymer U. [Diagnosis and initial management of acute myocardial infarction]. MMW Fortschr Med. (2019) 161:34–6. doi: 10.1007/s15006-019-0223-3 30830611

[18] Chen HY Li MC Liao D Li C Ji QM Guo P . The effect of computer-assisted cognitive remediation therapy on cognitive function, social function and quality of life in patients with vascular dementia. J Multidiscip Healthc. (2022) 15:2473–9. doi: 10.2147/JMDH.S378079 36324875 PMC9620993

[19] Zhou H Zhang J Li Y. The effects of high-quality nursing accompaniment on the sleep quality, negative psychological moods and quality of life of patients with acute myocardial infarct. Front Cardiovasc Med. (2025) 12:1562236. doi: 10.3389/fcvm.2025.1562236 40698004 PMC12279723

[20] Olatunji BO Deacon BJ Abramowitz JS Tolin DF. Dimensionality of somatic complaints: factor structure and psychometric properties of the Self-Rating Anxiety Scale. J Anxiety Disord. (2006) 20:543–61. doi: 10.1016/j.janxdis.2005.08.002 16198532

[21] Philippot A Dubois V Lambrechts K Grogna D Robert A Jonckheer U . Impact of physical exercise on depression and anxiety in adolescent inpatients: a randomized controlled trial. J Affect Disord. (2022) 301:145–53. doi: 10.1016/j.jad.2022.01.011 35007642

[22] Rodríguez-Herrera C López-Jiménez JJ Del Toro-Valero A Torres-Carrillo NM Torres-Carrillo N Godínez-Peña CA . The Newcastle satisfaction with nursing scales in a Mexican Oncology Hospital. Afr Health Sci. (2021) 21:60–6. doi: 10.4314/ahs.v21i1.10 34394282 PMC8356602

[23] Yiadom MYAB Gong W Bloos SM Bunney G Kabeer R Pasao MA . Shorter door-to-ECG time is associated with improved mortality in STEMI patients. J Clin Med. (2024) 13:2650. doi: 10.3390/jcm13092650 38731180 PMC11084706

[24] De Luca G Suryapranata H Ottervanger JP Antman EM. Time delay to treatment and mortality in primary angioplasty for acute myocardial infarction. Circulation. (2004) 109:1223–5. doi: 10.1161/01.CIR.0000121424.76486.20 15007008

[25] Xie H Gao M Lin Y Yi Y Liu Y. An emergency nursing and monitoring procedure on cognitive impairment and neurological function recovery in patients with acute cerebral infarction. NeuroRehabilitation. (2022) 51:161–70. doi: 10.3233/NRE-210310 35527573

[26] Anderson JL Morrow DA. Acute myocardial infarction. N Engl J Med. (2017) 376:2053–64. doi: 10.1056/NEJMra1606915 28538121

[27] Saleh M Ambrose JA. Understanding myocardial infarction. F1000Research. (2018) 7: 1378. doi: 10.12688/f1000research.15096.1 30228871 PMC6124376

[28] Murphy A Goldberg S. Mechanical complications of myocardial infarction. Am J Med. (2022) 135:1401–9. doi: 10.1016/j.amjmed.2022.08.017 36075485

[29] Lou X Xu H. The effectiveness of an emergency department nursing intervention on psychological symptoms and self-care capacities. Medicine. (2021) 100:e24763. doi: 10.1097/MD.0000000000024763 34032691 PMC8154481

