# Supplementary material for: Emergency procedural pathway combined with graded zoning management is associated with improved in-hospital survival and quality of life in patients with acute myocardial infarction
Source: Front Med (Lausanne). 2026 Jul 6;13:1841718. doi: 10.3389/fmed.2026.1841718 (PMC13381752; doi:10.3389/fmed.2026.1841718)
Supplement: Supplementary file 1 [file Table_1.docx]

**Supplementary Table S1. Baseline demographic and clinical characteristics by group.**

| Characteristic | Intervention group (IG, n = 62) | Control group (CG, n = 64) | Test statistic | *p* |
| --- | --- | --- | --- | --- |
| Age (years), mean ± SD | 56.30 ± 4.15 | 56.42 ± 4.38 | *t* = 0.16 | 0.873 |
| Male sex, n (%) | 36 (58.1) | 38 (59.4) | χ² = 0.02 | 0.881 |
| Body mass index (kg/m²), mean ± SD | 24.88 ± 2.20 | 24.90 ± 2.18 | *t* = 0.05 | 0.959 |
| Onset-to-presentation time (h), mean ± SD | 2.03 ± 0.70 | 2.06 ± 0.58 | *t* = 0.26 | 0.793 |
| Killip class, n (%) |  |  | χ² = 0.46 | 0.499 |
| – Class II | 40 (64.5) | 38 (59.4) |  |  |
| – Class III | 22 (35.5) | 26 (40.6) |  |  |
| Coronary heart disease, n (%) | 40 (64.5) | 42 (65.6) | χ² = 0.02 | 0.895 |
| Hypertension, n (%) | 22 (35.5) | 31 (48.4) | χ² = 2.16 | 0.142 |
| Diabetes mellitus, n (%) | 29 (46.8) | 33 (51.6) | χ² = 0.29 | 0.591 |
| STEMI, n (%) | 52 (83.9) | 53 (82.8) | χ² = 0.03 | 0.870 |
| NSTEMI, n (%) | 10 (16.1) | 11 (17.2) |  |  |
| Primary PCI, n (%) | 40 (64.5) | 42 (65.6) | χ² = 0.02 | 0.895 |
| Thrombolysis, n (%) | 22 (35.5) | 22 (34.4) |  |  |

Comorbidities are not mutually exclusive. CI, confidence interval; SD, standard deviation.

**Supplementary Table S2. Adjusted (multivariable) analyses for the primary outcome and MACE.**

| Outcome | Unadjusted estimate (95% CI) | Unadjusted *p* | Adjusted estimate (95% CI) ᵃ | Adjusted *p* |
| --- | --- | --- | --- | --- |
| In-hospital survival to discharge (IG vs CG); odds ratio | 6.92 (1.48 to 32.4) | 0.008 (Fisher) | 6.50 (1.32 to 32.0) | 0.022 |
| MACE (IG vs CG); odds ratio | 0.30 (0.09 to 0.99) | 0.042 (Fisher) | 0.30 (0.09 to 0.97) | 0.045 |

ᵃ Multivariable logistic regression adjusted for age, sex, body mass index, Killip class (II vs III), and the principal comorbidities (coronary heart disease, hypertension, diabetes mellitus). The number of events is small relative to the number of covariates; adjusted estimates should therefore be interpreted as supportive sensitivity analyses rather than as definitive causal estimates. CI, confidence interval; MACE, major adverse cardiovascular events.

**Supplementary Table S3. Disaggregated reperfusion timing metrics by treatment modality.**

| Metric | IG, mean ± SD (n) | CG, mean ± SD (n) | Mean difference (95% CI) | *p* |
| --- | --- | --- | --- | --- |
| Door-to-balloon time (min), among patients receiving primary PCI | 78.5 ± 12.3 (n = 40) | 92.4 ± 15.6 (n = 42) | −13.9 (−20.0 to −7.8) | < 0.001 |
| Door-to-needle time (min), among patients receiving thrombolysis | 32.5 ± 6.8 (n = 22) | 41.2 ± 8.9 (n = 22) | −8.7 (−13.5 to −3.9) | < 0.001 |
| Time to reperfusion (min), all reperfused patients (PCI or thrombolysis) | 56.4 ± 8.2 (n = 62) | 67.8 ± 11.5 (n = 64) | −11.4 (−14.9 to −7.9) | < 0.001 |

CI, confidence interval; PCI, percutaneous coronary intervention.
